# Inflammaging: Immune–Metabolic Crosstalk Between the Prostate–Testis and Musculoskeletal System

**DOI:** 10.3390/ijms27083612

**Published:** 2026-04-18

**Authors:** Sabrina Bossio, Daniele La Russa, Vittoria Rago, Michele Di Dio, Antonio Aversa, Anna Perri

**Affiliations:** 1 Department of Experimental and Clinical Medicine, Magna Graecia University of Catanzaro, 88100 Catanzaro, Italy; 2 Department of Biology, Ecology and Earth Sciences, University of Calabria, 87036 Rende, Italy; daniele.larussa@unical.it; 3 Department of Pharmacy, Health and Nutritional Sciences, University of Calabria, 87036 Rende, Italy; vittoria.rago@unical.it (V.R.); michele.didio@unical.it (M.D.D.); anna.perri@unical.it (A.P.); 4 Department of Surgery, Division of Urology, Annunziata Hospital, 87100 Cosenza, Italy; 5 Endocrine and Metabolic Diseases Unit, Annunziata Hospital, 87100 Cosenza, Italy

**Keywords:** inflammaging, prostate cancer, testicular dysfunction, epigenetic, sarcopenia, caloric restriction, nutraceuticals

## Abstract

Male reproductive aging is increasingly recognized as a systemic process in which inflammaging drives progressive dysfunction of urogenital tissues. Key mechanisms include immune–metabolic alterations, activation of the NOD-like receptor protein 3 (NLRP3) inflammasome, as well as epigenetic remodeling. Evidence from experimental and clinical studies suggests that these processes are often investigated independently, and integrative models in humans remain limited. Here, we propose a conceptual framework linking the prostate, testis, and skeletal muscle, in which oxidative stress may act as a mediator amplifying systemic dysregulation at different levels during the aging process. Lifestyle and metabolic interventions, including caloric restriction, resistance exercise, and selected nutraceuticals, may act as key modulators of inflammaging pathways, thus highlighting new potential targets for precision medicine approaches.

## 1. Introduction

### 1.1. Systemic Inflammaging and Male Reproductive System

The prostate and testis are highly sensitive to systemic inflammaging, a chronic low-grade inflammatory state characterized by oxidative stress, immune dysregulation, and metabolic dysfunction [1,2,3]. These processes converge on shared molecular pathways, including inflammasome activation, mitochondrial dysfunction, and impaired autophagy, which progressively affect male reproductive function during aging [4,5,6]. Together, these mechanisms position inflammaging as a unifying driver linking prostatic and testicular dysfunction within systemic aging. In this context, semen has emerged as a highly sensitive biomarker of environmental and systemic health, capable of reflecting early alterations induced by external stressors. Notably, conventional semen analysis parameters may not fully capture subtle molecular changes. In contrast, advanced approaches have revealed pollutant-associated alterations in oxidative stress, inflammatory signaling, and epigenetic regulation in spermatozoa, even in the absence of overt changes at the conventional level [7]. These findings are consistent with evidence showing that environmental pollutants, including particulate matter and endocrine-disrupting chemicals, can directly impair spermatozoa through oxidative stress-driven inflammatory and epigenetic mechanisms, often preceding detectable alterations in standard semen parameters.

### 1.2. Inter-Organ Crosstalk and the Prostate–Testis Axis

Building on this concept, inflammatory and metabolic alterations within the male reproductive organs extend beyond local tissue compartments, and they actively participate in systemic crosstalk with other aging-sensitive tissues, contributing to broader phenotypes such as sarcopenia and metabolic decline [8,9]. This supports the existence of an integrated prostate–testis axis embedded within whole-body aging networks. In this framework, reproductive, endocrine, immune, and metabolic pathways interact as a coordinated system rather than an isolated processes. This perspective moves beyond conventional hormone-centric models of male reproductive aging focused on androgen decline and hypothalamic-pituitary-gonadal (HPG) axis alterations [10,11]. Instead, this model identifies the interplay between inflammatory, metabolic, and immune pathways as key drivers of reproductive aging.

### 1.3. Emerging Therapeutic Perspectives and Epigenetic Regulation

Conventional therapies for male reproductive aging primarily target hormonal replacement but often overlook upstream inflammatory and metabolic mechanisms. This limitation has prompted growing interest in metabolism-targeting and non-conventional interventions aimed at modulating chronic inflammation and mitochondrial function [12,13].

In this model, oxidative stress should not be viewed as an isolated pathogenic factor, but as a functional component of systemic inflammaging that both sustains immune and metabolic dysregulation through self-reinforcing feedback loops [14,15]. This perspective highlights shared molecular drivers linking prostate and testicular dysfunction with systemic manifestations such as sarcopenia and metabolic decline. Notably, many of these alterations converge on epigenetic remodeling, which acts as a stable interface through which systemic inflammaging can exert long-term effects on gene regulation and tissue function [16]. The role of epigenetic remodeling as a unifying mechanistic layer linking inflammaging and the reproductive system will be discussed in detail in a dedicated section later in this review.

In this review, we provide a systems-level view of male reproductive aging, positioning inflammaging as a central framework that links local tissue remodeling to whole-body aging phenotypes. We also discuss how lifestyle and non-conventional interventions targeting shared inflammatory, metabolic, redox, and epigenetic pathways may represent promising strategies to preserve male reproductive health.

## 2. Hallmarks of Systemic Inflammaging

### 2.1. Conceptual Framework of Inflammaging

Inflammaging is a chronic, sterile inflammatory state driven by endogenous molecular damage rather than external pathogens. It is sustained by age-related immunosenescence, impaired cellular clearance, and defective resolution of inflammation. Within the broader framework of the hallmarks of aging, inflammaging is increasingly recognized as an integrated phenotype arising from the interplay among several key biological processes, including cellular senescence, mitochondrial dysfunction, and altered intercellular communication [17]. Originally conceptualized by Franceschi and colleagues, inflammaging is now widely recognized as a major driver of age-associated functional decline and increased susceptibility to chronic diseases, including cancer, metabolic disorders, neurodegeneration, and endocrine dysfunction [1]. Unlike acute inflammatory responses, inflammaging is triggered by endogenous danger signals that accumulate during aging as a result of progressive cellular and molecular damage. These signals, together with immune surveillance and defective resolution mechanisms, sustain persistent low-grade inflammatory state [4].

### 2.2. Oxidative Stress and Redox Signaling in Inflammation

Oxidative stress represents a central driver of inflammaging, in which reactive oxygen species (ROS) act as signaling molecules that amplify inflammatory pathways through self-reinforcing feed-forward loops [18,19]. Sustained ROS production contributes to a persistent inflammatory pathways through self-reinforcing feed forward loops [20,21]. Importantly, oxidative stress should not be viewed as an isolated mechanism, but rather as a coordinating component within a broader network that integrated immunosenescence, metabolic dysregulation, and inflammasome activation.

### 2.3. Inflammatory Signaling Networks: NF-κB, STAT3, and NLRP3

Consistent with the central role of oxidative stress in inflammaging, redox-sensitive signaling cascades engage key inflammatory pathways, including nuclear factor kappa B (NF-κB), mitogen-activated protein kinases (MAPKs), and the NOD-like receptor protein 3 (NLRP3) inflammasome, promoting the production of pro-inflammatory cytokines such as interleukin-1 beta (IL-1β), interleukin-6 (IL-6), and tumor necrosis factor-alpha (TNF-α) [18,19,22].

**Figure 1:**
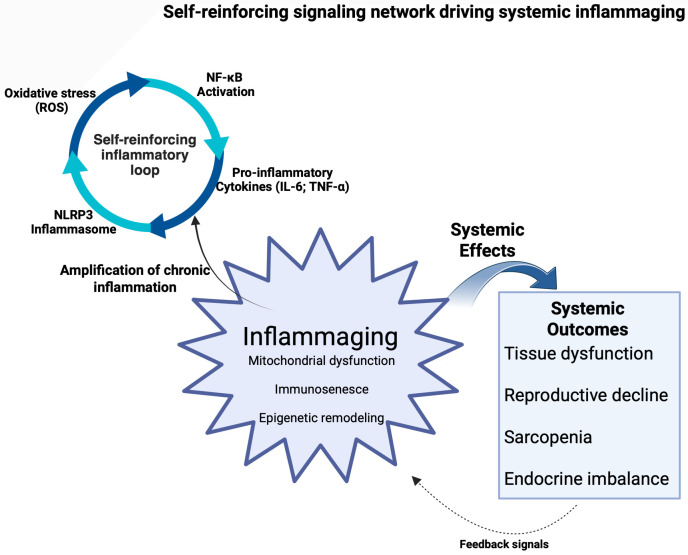
Self-reinforcing signaling network driving systemic inflammaging. Schematic representation of the signaling network underlying inflammaging. Oxidative stress-derived reactive oxygen species (ROS) activate redox-sensitive pathways, including nuclear factor kappa B (NF-κB), promoting the production of pro-inflammatory cytokines (e.g., interleukin-6 (IL-6), tumor necrosis factor alpha (TNF-α)). These cytokines further amplify inflammatory signaling and activate the NOD-like receptor protein 3 (NLRP3) inflammasome, establishing a self-reinforcing loop that sustains chronic low-grade inflammation. This process drives systemic inflammaging, characterized by mitochondrial dysfunction, immunosenescence, and epigenetic remodeling, ultimately leading to tissue damage, reproductive decline, sarcopenia, and endocrine imbalance. Arrows indicate the direction of signaling flow and feedback mechanisms within the inflammatory network. Created in BioRender. Bossio, S. (2026) https://BioRender.com/veju96g (accessed on 2 April 2026).

These pathways operate within an interconnected signaling network in which ROS-mediated NF-κB activation induces cytokine production, which further amplifies inflammatory signaling through signal transducer and activator of transcription 3 (STAT3) and NLRP3 inflammasome activation, thereby sustaining chronic inflammatory responses during aging (Figure 1).

Importantly, this network represents a self-reinforcing system in which redox balance acts a central coordinating node sustaining inflammatory signaling through positive feedback mechanisms. Aging-associated immune remodeling contributes to elevated circulating levels of inflammatory biomarkers, including TNF-α, IL-6, and C-reactive protein (CRP), which correlate with frailty and tissue dysfunction [23].

### 2.4. Immunosenescence and Systemic Inflammatory Remodeling

Inflammaging is sustained by several interconnected age-associated processes, including immunosenescence, accumulation of senescent cells, impaired resolution of inflammation, and metabolic dysregulation. Age-related immune remodeling, thymic involution, and the emergence of senescence-associated secretory phenotype (SASP) factors contribute to the persistence of chronic low-grade inflammation and reinforce systemic immune–metabolic imbalance during aging [24,25]. Rather than isolated organ-specific alterations, systemic inflammaging emerges from the integration of multiple biological processes, including oxidative stress, impaired proteostasis, defective autophagy, and inflammasome activation, which collectively disrupt cellular homeostasis and sustain chronic inflammatory signaling [26]. Importantly, these systemic inflammatory processes also affect tissues of the male reproductive system. The prostate and the testis are particularly sensitive to chronic low-grade inflammation, immune remodeling, and metabolic imbalance. Within these organs, inflammaging-related mechanisms such as oxidative stress, inflammasome activation, impaired autophagy, and immune dysregulation contribute to tissue remodeling, endocrine dysfunction, and progressive reproductive decline. Taken together, these findings support a model in which inflammaging is driven by an integrated network linking redox imbalance, immune activation, and metabolic dysfunction, ultimately shaping tissue-specific vulnerability within the male reproductive system.

**Figure 2:**
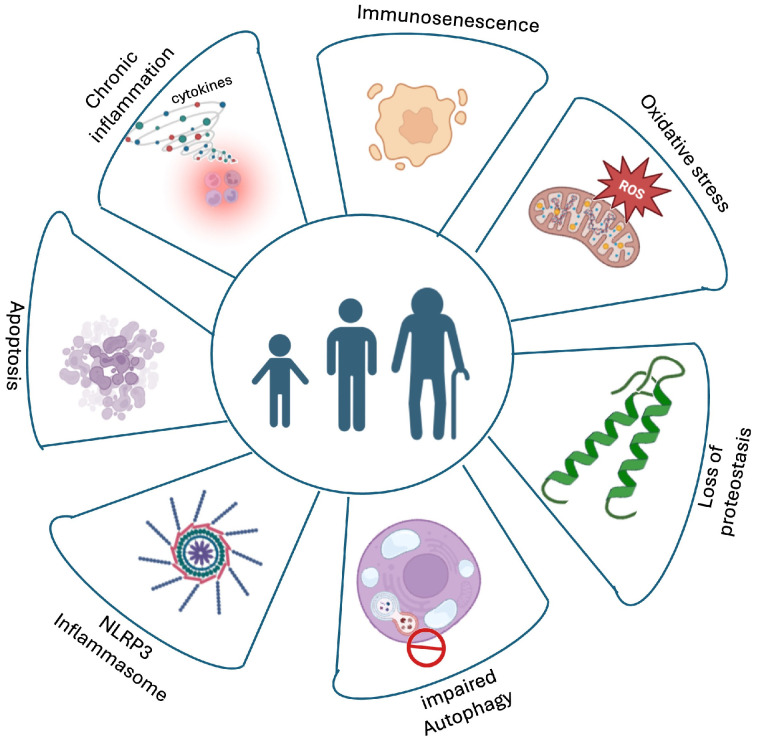
Hallmarks of inflammaging: Schematic representation of the major biological processes underlying systemic inflammaging, including chronic low-grade inflammation, immunosenescence, oxidative stress, proteostasis loss, impaired autophagy, inflammasome activation, and apoptosis. These interconnected mechanisms contribute to functional decline across multiple tissues, including the prostate, testis, and skeletal muscle, promoting age-related reproductive and systemic dysfunctions. Created in BioRender. Bossio, S. (2026) https://BioRender.com/br9lpq3 (accessed on 14 February 2026).

A schematic overview of the main mechanisms underlying inflammaging and their role in male reproductive and systemic aging is illustrated in Figure 2.

### 2.5. Prostatic Inflammaging: Chronic Inflammation and Prostatic Tissue Remodeling

Within the male reproductive system, inflammaging disrupts tissue homeostasis, promoting structural remodeling, altered cellular signaling, and increased disease susceptibility. The prostate gland is particularly vulnerable to age-related inflammatory processes, which can be triggered by microbial infections, chemical injury, dietary factors, hormonal imbalances, and age-related immune dysregulation [27,28]. Chronic inflammation is increasingly recognized as a key contributing mechanism underlying several prostate disorders [2,3].

Persistent immune cell infiltration and sustained cytokine production within prostatic tissue promote structural and functional alterations that progressively impair prostate homeostasis during aging [29]. While benign prostatic hyperplasia (BPH) and prostate cancer are discussed as clinically relevant outcomes, the primary focus of this section is on chronic prostatic inflammation as a central driver of tissue remodeling during aging.

Among these conditions, BPH represents a common model of inflammation-associated tissue remodeling within the broader framework of inflammaging [30]. In this context, androgen signaling provides a permissive hormonal background, while chronic inflammation synergizes with growth factor and amplifies proliferative and remodeling processes during aging [31,32,33,34]. Aging is also associated with oxidative imbalance, immune dysregulation, and metabolic alteration within prostatic tissue [14,35]. Increased ROS production, impaired antioxidant defenses, and metabolic stress activate pro-inflammatory pathways, including NF-κB signaling and inflammasome activation, thereby sustaining a chronic inflammatory microenvironment and promoting stromal remodeling [14,35,36,37,38]. Furthermore, Chronic oxidative stress also promotes cellular senescence with SASP-associated factors acting as amplifiers of stromal-epithelial remodeling and inflammatory signaling within the aging prostate [39].

Beyond benign remodeling, inflammaging contributes to prostate cancer (PCa) development by promoting genomic instability and activation of pro-survival and proliferative signaling pathways [27,28].

Proliferative inflammatory atrophy (PIA) has been proposed as a potential link between chronic inflammatory injury and prostate carcinogenesis [40,41], while inflammatory cytokines such as IL-6, interleukin-17 (IL-17), and TNF-α activate NF-κB and STAT3 signaling and interact with androgen receptor pathways to promote epithelial survival and proliferation.

**Figure 3:**
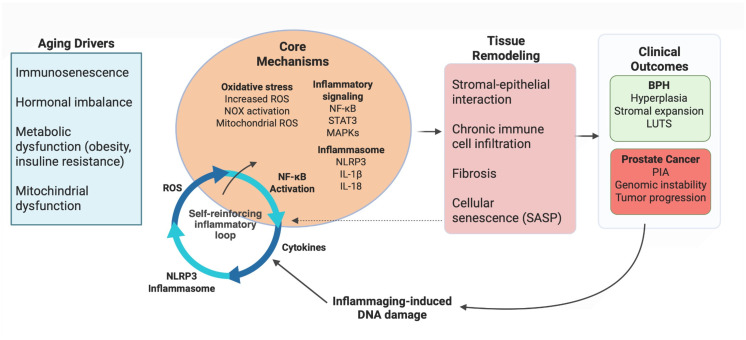
Self-reinforcing inflammatory network driving prostatic inflammaging and disease progression. Schematic representation of the mechanisms linking aging-associated systemic drivers to prostatic tissue remodeling and disease outcomes. Immunosenescence, hormonal imbalance, and metabolic dysfunction promote oxidative stress and activation of inflammatory pathways (e.g., nuclear factor kappa B (NF-κB), signal transducer and activator of transcription 3 (STAT3), mitogen-activated protein kinases (MAPKs)) and the NOD-like receptor protein 3 (NLRP3) inflammasome, establishing a chronic self-sustaining inflammatory state. In the prostate, this environment drives stromal-epithelial remodeling, immune cell infiltration, fibrosis, and cellular senescence associated with the senescence-associated secretory phenotype (SASP), further amplifying inflammatory signaling. These alterations contribute to disease progression, linking inflammaging to benign prostatic hyperplasia (BPH), lower urinary tract symptoms (LUTS), proliferative inflammatory atrophy (PIA), and prostate cancer initiation and progression. Arrows indicate the direction of signaling pathways and feedback interactions within the inflammatory network. Created in BioRender. Bossio, S. (2026) https://BioRender.com/416u1y1 (accessed on 2 April 2026).

Within this framework, oxidative stress acts as a mechanistic bridge between chronic inflammation and malignant transformation. ROS activate transcription factors such as NF-κB, STAT3, and hypoxia-inducible factor 1-alpha (HIF-1α) [42,43,44], sustaining inflammation, angiogenesis, and metabolic adaptation. In this context, nuclear factor erythroid 2-related factor 2 (Nrf2) exhibits a context-dependent role: while transient activation may exert tumor-suppressive effects during carcinogenesis, persistent activation promotes metabolic reprogramming, tumor progression, and therapy resistance [45,46,47,48,49,50]. Collectively, these findings support a model in which inflammaging acts as a unifying framework linking chronic inflammatory remodeling to both BPH and malignant outcomes, with NLRP3 inflammasome signaling emerging as a key molecular axis connecting aging, inflammation, and prostatic disease progression (Figure 3) [51,52].

In the male reproductive system, the prostate and the testis represent organ-specific targets of systemic and local inflammaging, in which shared hallmarks of aging, including chronic low-grade inflammation, immune remodeling, oxidative stress, and endocrine dysregulation, drive divergent pathological outcomes, ranging from benign and malignant prostatic remodeling to progressive spermatogenic decline.

### 2.6. Testicular Inflammaging: Immune–Metabolic and Endocrine Dysregulation

Male aging is characterized by progressive testicular decline associated with the interplay of oxidative stress, mitochondrial dysfunction, inflammatory signaling, immunosenescence, impaired autophagy, apoptosis, and endocrine dysregulation [3]. However, whether inflammatory remodeling represents a primary driver of testicular aging or a secondary consequence of systemic alterations remains debated, as these processes appear to be closely interconnected. At the neuroendocrine level, age-related alterations in hypothalamic gonadotropin-releasing hormone (GnRH) pulsatility reduce luteinizing hormone (LH) and follicle-stimulating hormone (FSH) secretion, impairing testosterone synthesis by Leydig cells and Sertoli cell-mediated support of spermatogenesis. These alterations contribute to late-onset hypogonadism (LOH), characterized by reduced testosterone bioavailability and impaired reproductive function [53]. At the tissue level, aging testes display structural and functional alterations, degeneration of the seminiferous epithelium, germ cell loss, fibrosis, and vascular impairment, reflecting cumulative oxidative damage and impaired proteostasis [54,55]. Leydig cells exhibit reduced expression of key steroidogenic enzymes (CYP11A1, StAR, 3β-HSD) and mitochondrial dysfunction, while Sertoli cells show downregulation of junctional proteins such as ZO-1 and occludin, leading to blood-testis barrier (BTB) disruption and loss of immune privilege [56]. These changes are accompanied by immune remodeling characterized by T-cell exhaustion, reduced regulatory T-cell activity, and a shift toward pro-inflammatory M1 macrophage polarization, collectively promoting a chronic inflammatory microenvironment [57,58].

Within the inflammaging context, excessive ROS generation acts as a reinforcing mechanism linking immune dysregulation to mitochondrial dysfunction and germ-cell loss. Oxidative stress overwhelms antioxidant defenses, contributing to cellular damage and inflammatory signaling [15]. In addition, cellular senescence of Leydig, Sertoli, and endothelial cells, which contributes to local inflammation through p38 mitogen-activated protein kinase (p38 MAPK) signaling and the acquisition of a SASP that reinforces local inflammation [59].

Metabolic dysregulation further reinforces this immune–metabolic axis as alterations in mitochondrial metabolism and energy homeostasis promote oxidative stress and activate inflammasome-dependent inflammatory pathways, thereby amplifying cytokine production and macrophage activation [15,58].

Emerging evidence identifies the NLRP3 inflammasome signaling as a key molecular mediator of testicular inflammaging. NLRP3 is expressed in multiple testicular cell types and contributes to immune regulation, steroidogenesis, and maintenance of tissue homeostasis [56,59]. Increased NLRP3 activation has also been observed in pathological conditions such as obesity, varicocele, and infection, where it correlates with impaired sperm quality and inflammatory cytokine production [60,61]. Nevertheless, testicular inflammaging is unlikely to be exclusively driven by inflammasome activation. In addition, to inflammasome activation, ROS-mediated signaling, p38 MAPK activation, cytokine secretion, and immune cell infiltration following BTB disruption further contribute to the establishment of a chronic inflammatory microenvironment [15,58,59]. Importantly, the relationship between systemic inflammaging and testicular dysfunction appears to be bidirectional as a systemic inflammatory and metabolic alterations promote testicular aging, while declining Leydig cell function and reduced testosterone bioavailability may in turn exacerbate systemic metabolic and endocrine dysregulation [52]. These alterations translate into clinically measurable outcomes, including reduced semen quality and declining circulating testosterone levels, which are commonly used to define male reproductive aging and LOH [53,62,63].

**Figure 4:**
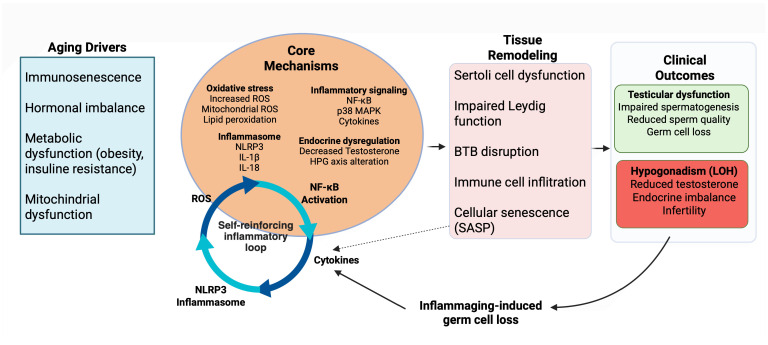
Self-reinforcing inflammatory network underlying testicular inflammaging and dysfunction. Immunosenescence, hormonal imbalance, metabolic dysfunction, and mitochondrial alterations promote oxidative stress and inflammatory signaling (e.g., nuclear factor kappa B (NF-κB), p38 mitogen-activated protein kinase (p38 MAPK)), along with activation of the NOD-like receptor protein 3 (NLRP3) inflammasome, establishing a self-reinforcing inflammatory loop. In the testis, these processes impair Sertoli and Leydig cell function and disrupt blood-testis barrier (BTB) integrity, and promote cellular senescence associated with the senescence-associated secretory phenotype (SASP), further amplifying inflammation. This altered microenvironment compromises spermatogenesis, leading to germ cell loss and reduced sperm quality. These changes ultimately result in late-onset hypogonadism (LOH), endocrine imbalance, and infertility. Arrows indicate the direction of signaling pathways and feedback interactions within the inflammatory network. Created in BioRender. Bossio, S. (2026) https://BioRender.com/lx396fb (accessed on 2 April 2026).

Collectively, these findings support a model in which testicular inflammaging is driven by an integrated, self-reinforcing network linking oxidative stress, immune remodeling, endocrine dysregulation, and metabolic dysfunction, ultimately leading to germ cell loss and reproductive decline (Figure 4).

## 3. Epigenetic Remodeling in Male Reproductive Inflammaging: Molecular Memory and Metabolic Crosstalk

Emerging evidence suggests that age-related epigenetic alterations may contribute to the transgenerational transmission of reproductive aging risk [64]. Aging and inflammaging induce persistent alterations in gene regulation that extend beyond transient cytokine signaling, positioning epigenetic mechanisms as key modulators of cellular identity, inflammatory memory, and disease susceptibility within the male reproductive system [16]. These mechanisms, including DNA methylation, histone post-translational modifications, non-coding RNA regulation, and chromatin remodeling, enable stable reprogramming of gene expression without altering DNA sequence, thereby sustaining inflammatory and stress-responsive transcriptional states over time [65]. Genome-wide epigenetic biomarkers of aging, reflecting cumulative DNA methylation drift, correlate positively with systemic inflammatory burden, supporting a direct link between chronic inflammation and epigenetic dysregulation during senescence [66,67]. Importantly, a distinction should be made between age-associated epigenetic drift and active epigenetic reprogramming. While epigenetic drift reflects cumulative molecular damage and serves as a biomarker of biological aging, active reprogramming involves inflammatory and metabolic signals that reshape chromatin organization and gene expression, ultimately contributing to disease susceptibility and tissue dysfunction.

### 3.1. Epigenetic Mechanisms Linking Inflammaging and Prostate

In the prostate, epigenetic remodeling emerges as a critical mechanism linking inflammaging to benign and malignant disease susceptibility. Both BPH and prostate cancer display distinct DNA methylation and hydroxymethylation profiles compared with normal tissue, suggesting inflammation-driven epigenetic reprogramming in response to aging-related stress [68]. Aberrant promoter hypermethylation of tumor suppressor genes (e.g., *GSTP1, RARβ2, RASSF1A*), together with global DNA hypomethylation, has been described with advancing age and in prostate cancer, contributing to genomic instability and neoplastic predisposition [69,70]. Histone modifications and changes in chromatin accessibility, together with DNA methylation, integrate inflammatory signals mediated by pathways such as NF-κB and STAT3, stabilizing pro-inflammatory transcriptional programs and potentially altering androgen receptor binding dynamics in both BPH and PCa contexts [71]. Non-coding RNAs, including miR-21, miR-146a, and miR-155, further modulate inflammatory signaling and epigenetic memory, reinforcing persistent low-grade inflammation in aging prostate tissues [65].

### 3.2. Epigenetic Alterations in Testicular Aging and Spermatogenesis

Similar inflammaging-driven epigenetic mechanisms operate in the testis, where they directly impair germ cell integrity, spermatogenesis, and reproductive fitness. Aging-associated DNA methylation drift affects both germ and somatic cells, characterized by hypomethylation of repetitive elements and locus-specific hypermethylation at genes involved in spermatogenesis, DNA repair, and steroidogenesis, correlating with reduced sperm quality and increased DNA fragmentation [64,72]. Chronic inflammation and oxidative stress interfere with the tightly regulated histone modification landscape required for spermatogenesis, leading to aberrant histone retention, altered Histone H3 lysine 9 (H3K9) and Histone H3 lysine 27 (H3K27) methylation, and impaired chromatin compaction in spermatozoa [15]. Age-dependent dysregulation of non-coding RNAs, including miR-21, miR-34a, and miR-146a, further perturbs pathways governing germ cell survival, Sertoli cell function, and immune regulation [64]. Importantly, epigenetic alterations in the aging testis may exert effects beyond local dysfunction, as inflammatory and metabolic stressors can induce stable epigenetic marks in sperm that influence offspring development and disease susceptibility, implicating testicular inflammaging in the transgenerational transmission of aging-associated phenotypes [72,73]. However, it should be noted that most direct evidence for transgenerational epigenetic inheritance is largely derived from preclinical models. In contrast, evidence in humans remains limited and is largely based on associative observations.

### 3.3. Metabolic Regulation of Epigenetic Remodeling

Beyond the classical epigenetic mechanisms, growing evidence indicates that epigenetic remodeling is tightly integrated with cellular metabolism and nutrient availability. Recent studies demonstrate that epigenetic enzyme activity is strongly influenced by metabolic intermediates generated through nutrient-dependent pathways, which act as essential substrates or cofactors for epigenetic modifications [74]. For instance, acetyl-CoA acts as a key donor for histone acetylation reactions, whereas S-adenosyl-methionine (SAM) provides the methyl groups required for DNA and histone methylation, thereby directly linking cellular metabolism to epigenetic regulation [75]. Within this framework, nutritional status and metabolic fluctuations profoundly influence inflammatory gene expression by modulating the epigenetic landscape. Excess nutrient availability and metabolic dysregulation promote epigenetic activation of pro-inflammatory pathways, including NF-κB-dependent transcriptional signaling, while chronic inflammation further reinforces epigenetic instability through DNA methylation drift and altered histone modification [74,76]. In addition, excess nutrient intake compromises autophagy through the synergistic effects of elevated acetyl-CoA levels, overactivation of mechanistic target of rapamycin (mTOR), and dysregulation of key autophagy-related proteins [77]. The resulting autophagic deficiency facilitates the accumulation of damaged organelles and pathological accumulation and sustained activation of sequestosome 1 (p62) [74,78].

### 3.4. Nutritional and Metabolic Modulation of the Epigenome

Low-calorie diets may mitigate aging and inflammation-related pathologies, including cancer, by modulating chromatin remodeling and epigenetic mechanisms via diet-dependent metabolites. The balance of macronutrients and overall caloric intake can influence the progression of age-related diseases and the accumulation of molecular damage. These effects are mediated by nutrient-sensing pathways, including phosphoinositide 3-kinase (PI3K)/protein kinase B (AKT)-mTOR, which regulate metabolic signaling and reshape the epigenetic landscape [74]. This integrative view aligns with the concept proposed by Szarc vel Szic et al. (2015), which describes inflammaging not merely as a consequence of cumulative damage, but as a metabolically and epigenetically programmable process, in which dietary components modulate chromatin remodeling, DNA methylation, and inflammatory gene expression across the lifespan, thereby influencing healthy aging approaches and interindividual disease susceptibility [79].

Overall, these observations support the notion that epigenetic remodeling serves as a central molecular hub through which inflammatory, metabolic, and nutritional cues converge to shape aging trajectories in the male reproductive system. By integrating nutrient availability, metabolic signaling, and inflammatory stress, the epigenome acts as a dynamic and potentially reversible regulator of tissue vulnerability, reproductive decline, and transgenerational risk. This integrative framework provides a mechanistic basis for understanding how lifestyle- and metabolism-targeting interventions may modulate inflammaging-associated epigenetic memory and disease susceptibility.

This perspective positions epigenetic remodeling not merely as a downstream consequence of aging, but as a dynamic regulatory layer that stabilizes inflammaging-related signals and translates transient metabolic and inflammatory cues into long-term alterations in reproductive function.

## 4. Sarcopenia and Inflammaging: A Bidirectional Systemic Axis in Male Reproductive System

Sarcopenia is increasingly recognized as a systemic manifestation of inflammaging that shares common endocrine, metabolic, and inflammatory mechanisms with male reproductive aging, rather than representing an independent condition.

In this context, it is mechanistically linked to male reproductive aging through shared endocrine and inflammatory pathways, particularly those involving androgen signaling and systemic immune–metabolic regulation [80,81].

According to the European Working Group on Sarcopenia in Older People 2 (EWGSOP2), sarcopenia is defined by an age-related decline in skeletal muscle quantity and quality associated with impaired physical performance and increased morbidity and mortality in older individuals [82,83]. This condition frequently coexists with metabolic dysfunction, endocrine alterations, and chronic low-grade inflammation, which collectively promote muscle catabolism and impair regenerative capacity [8]. Accumulating evidence suggests that inflammaging may represent a major contributing factor to muscle decline rather than being solely an associated condition. Age-related immune activation, immunosenescence, adipose tissue dysfunction, and the release of damage-associated molecular patterns converge on sustained activation of inflammatory pathways, including NF-κB and Janus kinase (JAK)/signal transducer and activator of transcription (STAT) signaling, thereby disrupting skeletal muscle homeostasis and accelerating muscle wasting [8,84]. Notably, the relationship between inflammaging and sarcopenia is bidirectional, as muscle loss and reduced physical activity further exacerbate systemic inflammation and metabolic dysregulation, establishing a self-perpetuating vicious cycle [84]. Thus, sarcopenia should be viewed not merely as a downstream consequence of inflammaging but rather as a systemic co-driver that further amplifies inflammatory and metabolic dysregulation during aging.

In men, this condition is tightly interconnected with age-related endocrine decline, particularly progressive testicular dysfunction. Skeletal muscle expresses androgen receptors, and testosterone plays a central role in maintaining muscle mass, strength, and regenerative capacity by regulating satellite cell activity, protein synthesis, and inflammatory signaling [80,85,86].

**Figure 5:**
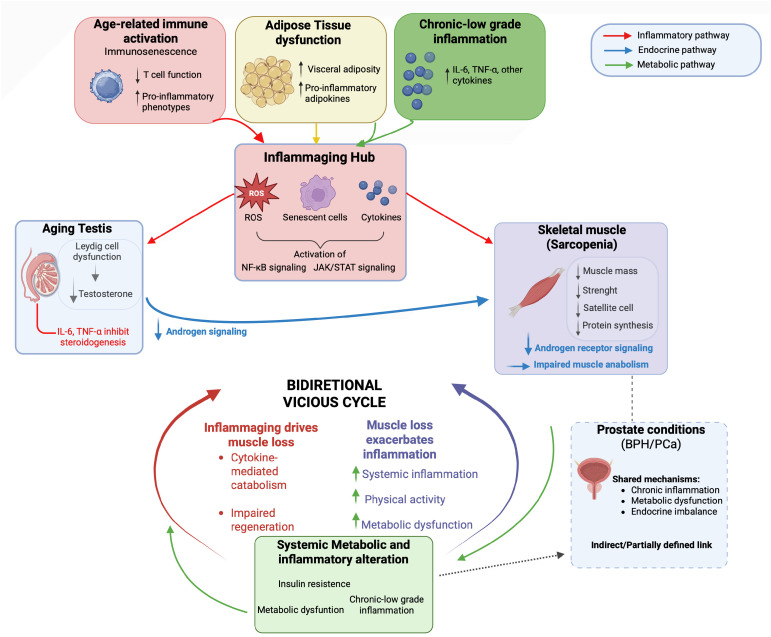
Proposed integrated mechanistic model: the vicious cycle of sarcopenia-male reproductive aging-inflammaging. Age-related immune activation, adipose tissue dysfunction, and chronic low-grade inflammation converge in an inflammaging hub characterized by increased ROS and pro-inflammatory cytokines (e.g., interleukin-6 (IL-6), tumor necrosis factor-alpha (TNF-α)), leading to activation of nuclear factor kappa B (NF-κB) and Janus kinase (JAK)/signal transducer and activator of transcription (STAT) signaling. These pathways impair Leydig cell steroidogenesis, resulting in reduced testosterone levels and decreased androgen receptor-mediated anabolic signaling in skeletal muscle. Sarcopenia both results from and contributes to inflammaging, as inflammation promotes muscle catabolism, while muscle loss and reduced physical activity exacerbate systemic inflammation and metabolic dysfunction, establishing a bidirectional vicious cycle. Systemic alterations further connect this network to prostate conditions through shared, although partially defined, mechanisms. Arrows indicate the direction of signaling interactions; red, blue, and green arrows represent inflammatory, endocrine, and metabolic pathways, respectively. Created in BioRender. Bossio, S. (2026) https://BioRender.com/85ykvpo (accessed on 2 April 2026).

This interplay positions skeletal muscle as a key peripheral effector of the HPG axis during aging linking testicular endocrine to musculoskeletal homeostasis. Age-related reductions in circulating testosterone levels are closely associated with sarcopenia development. Chronic low-grade inflammation negatively affects Leydig cell steroidogenesis, with elevated IL-6 and TNF-α levels inversely correlating with serum testosterone, reinforcing a self-sustaining axis linking inflammaging, hypogonadism, and muscle wasting [81]. Beyond testicular dysfunction, sarcopenia shares common pathogenic mechanisms with age-related prostatic conditions, including chronic inflammation, metabolic dysregulation, and endocrine imbalance, although these are considered here within a systemic framework rather than as primary outcomes. Direct mechanistic links between sarcopenia and BPH remain incompletely defined. Nevertheless, emerging evidence suggests that systemic metabolic and inflammatory alterations may contribute to both sarcopenia and prostatic diseases. Mendelian randomization analyses indicate that genetic determinants of basal metabolic rate and appendicular lean mass are linked to increased BPH susceptibility, supporting the concept that systemic metabolic and inflammatory alterations contribute to both conditions [87]. However, these associations may also reflect overlapping genetic and regulatory networks, as metabolic, inflammatory, and endocrine pathways frequently intersect in complex aging phenotypes. In this framework, sarcopenia and BPH may be viewed as parallel manifestations of a broader aging phenotype rather than isolated organ-specific disorders. In the context of prostate cancer, muscle loss has emerged as a clinically relevant manifestation of systemic inflammaging and endocrine disruption with important prognostic implications. Clinical studies and meta-analyses demonstrate that, this condition is highly prevalent among men with prostate cancer and is associated with poorer progression-free survival and increased non-cancer mortality [88,89]. In advanced and metastatic hormone-sensitive disease, sarcopenia independently predicts worse failure-free survival and earlier progression to castration resistance [90,91]. Importantly, androgen deprivation therapy accelerates muscle loss by inducing profound testosterone depletion, thereby exacerbating age-related sarcopenia and contributing to frailty and reduced treatment tolerance [92,93]. Collectively, these findings reinforce the view of sarcopenia as a systemic aging phenotype driven by inflammaging and endocrine dysregulation, linking musculoskeletal decline with age-related alterations of the male reproductive system, particularly hypogonadism and prostatic disease. Importantly, accumulating evidence indicates that resistance training and combined lifestyle interventions can partially reverse or attenuate age-related muscle loss, highlighting the plasticity of the musculoskeletal system and supporting the concept that sarcopenia represents a modifiable component of the inflammaging network [94]. These observations support the concept of an integrated prostate-testis-muscle axis, in which systemic inflammaging simultaneously affects reproductive and musculoskeletal health during aging (Figure 5).

## 5. Targeting Inflammaging and Sarcopenia in Male Reproductive System: Therapeutic and Lifestyle Strategies

Alongside conventional pharmacological approaches, non-conventional therapies and lifestyle interventions have gained increasing attention as strategies to mitigate age-related functional decline in the male reproductive system. At the molecular level, these interventions are increasingly recognized to act through epigenetic mechanisms linking metabolic and nutritional cues to inflammatory signaling, thereby shaping chromatin dynamics, mitochondrial function, and stress responses.

Within this framework, lifestyle- and metabolism-targeting strategies may modulate inflammaging by influencing epigenetic memory and core aging pathways rather than acting solely as symptomatic or antioxidant approaches [74,79]. These interventions converge on key hallmarks of aging, including redox imbalance, mitochondrial dysfunction, impaired autophagy, and chronic low-grade inflammation, thereby influencing both reproductive aging and systemic phenotypes such as sarcopenia [9]. Among non-conventional strategies, antioxidants and nutraceuticals represent the most extensively investigated approaches. Reduced antioxidant capacity and elevated ROS levels have been consistently reported in the seminal plasma of infertile and aging men, supporting a pathogenic role for redox imbalance in this context [95]. Accordingly, antioxidant supplementation has been shown to alleviate oxidative damage and improve reproductive parameters [96].

α-Lipoic acid (ALA), a mitochondrial dithiol compound, modulates redox balance and mitochondrial function, contributing to metabolic regulation [97,98]. Notably, ALA has been proposed as a caloric restriction mimetic, activating AMP-activated protein kinase (AMPK)- and sirtuin 1 (SIRT1)-dependent pathways, enhancing mitochondrial biogenesis, and promoting autophagic flux [99]. Experimental and clinical evidence indicate that ALA supplementation reduces seminal oxidative stress and improves sperm parameters, testosterone levels, and testicular hemodynamics, while restoring antioxidant capacity and limiting lipid peroxidation [100,101,102]. Although direct evidence on inflammasome modulation in the aging testis remains limited, available data support its role in regulating antioxidant and apoptotic pathways [103,104].

Similarly, coenzyme Q10 supplementation has demonstrated beneficial effects on sperm motility, mitochondrial activity, and antioxidant capacity in randomized controlled trials, reinforcing the central role of mitochondrial redox balance in male reproductive function [105]. Polyphenolic compounds, including quercetin, resveratrol, and curcumin, further exemplify nutraceutical strategies targeting inflammaging-related pathways. Quercetin exerts antioxidant and anti-inflammatory effects by modulating key pathways such as, NF-κB, PI3K/AKT, and mitochondrial ROS production, thereby attenuating testicular oxidative stress and apoptotic signaling [106,107,108]. Resveratrol activates sirtuin-dependent pathways, enhancing mitochondrial biogenesis, antioxidant defenses, and autophagic flux in testicular tissue [109]. Curcumin formulations inhibit NLRP3 inflammasome activation and restore AMPK/mTOR-mediated autophagy, preserving BTB integrity and spermatogenic potential [110]. However, it should be noted that a substantial proportion of the mechanistic evidence supporting nutraceutical interventions derives from preclinical studies, whereas robust randomized controlled trials in humans remain comparatively limited, warranting further investigation. Emerging evidence also implicates gut microbiota–derived metabolites in the regulation of reproductive aging. The phenolic metabolite 3-hydroxyphenylacetic acid (3-HPAA) has been shown to restore spermatogenic function in aged mice through glutathione peroxidase 4 (GPX4)-mediated inhibition of ferroptosis, highlighting the interplay between microbial metabolism, redox regulation, and male fertility [111].

Dietary interventions, together with supplementation, represent a cornerstone of lifestyle-based strategies targeting inflammaging and sarcopenia.

Caloric restriction (CR) has been shown to modulate the AMPK/SIRT1/mTOR axis, enhance autophagy and mitochondrial quality control, and suppress systemic inflammation, thereby improving testicular homeostasis and reproductive function during aging [112,113]. It also reduces NLRP3 inflammasome activation and fibrotic remodeling in the prostate [114]. However, excessive or prolonged CR may impair spermatogenesis and may be difficult to sustain in aging populations, requiring careful evaluation to avoid adverse effects such as malnutrition or frailty [115,116], underscoring the importance of metabolic balance

Lifestyle interventions specifically targeting sarcopenia, together with nutraceutical supplementation and caloric restriction, represent a crucial complementary strategy to counteract inflammaging in male reproductive aging. The main lifestyle-based strategies and their proposed molecular targets in the context of male reproductive aging are summarized in Table 1.

Regular physical activity, particularly resistance training, exerts potent anti-inflammatory and antioxidant effects, improving mitochondrial function and anabolic signaling while reducing circulating pro-inflammatory mediators. Evidence suggests that resistance training performed at moderate to high intensity and repeated several times per week can effectively stimulate anabolic signaling, improve muscle strength, and counteract age-related muscle loss [94].

Skeletal muscle acts as an endocrine organ, releasing myokines in response to exercise that modulate systemic inflammation and metabolic homeostasis, thereby contributing to inter-organ crosstalk relevant to reproductive aging [117,118]. Adequate protein intake is essential for the prevention and management of sarcopenia, particularly when combined with physical exercise. Essential amino acids or leucine supplementation enhances muscle protein synthesis, counteracts anabolic resistance, and may influence hormonal and metabolic pathways involved in male reproductive function [119]. Additional lifestyle-related factors, including optimization of vitamin D status, have been associated with improved muscle function and modulation of androgen levels. Together, these effects support an integrated lifestyle approach to counteract inflammaging and age-related dysfunctions of the testis and prostate [120,121], through shared inflammatory, metabolic, and endocrine pathways. Preclinical and clinical evidence suggest that combining caloric restriction with structured exercise may exert synergistic effects on metabolic and inflammatory regulation, improving insulin sensitivity, body composition, and systemic homeostasis more effectively than either intervention alone [122,123]. Although these studies were conducted in metabolic disease models, they provide proof-of-concept evidence that integrated lifestyle strategies can modulate shared metabolic and inflammatory pathways.

Future research may therefore investigate whether similar combinatorial approaches, such as CR, caloric restriction mimetics, or targeted antioxidant combined with resistance training, could beneficially influence inflammaging-related mechanisms underlying male reproductive aging.

Such multi-target strategies support an integrated prostate-testis-muscle axis, in which systemic inflammaging simultaneously affects reproductive and musculoskeletal health during aging.

**Figure 6:**
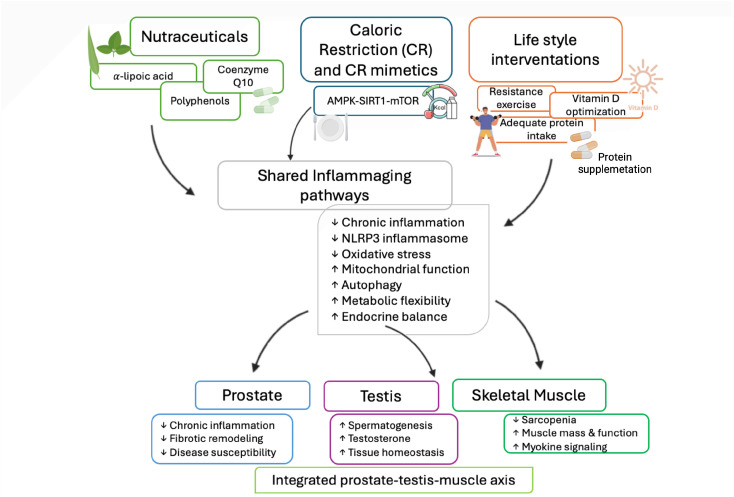
Lifestyle and integrative interventions targeting inflammaging in male reproductive aging. Nutraceuticals, caloric restriction and its mimetics, and lifestyle interventions converge on shared inflammaging pathways, including chronic inflammation, NOD-like receptor protein 3 (NLRP3) inflammasome activation, oxidative stress, mitochondrial dysfunction, impaired autophagy, and endocrine imbalance. By modulating these interconnected mechanisms, such interventions exert systemic effects on the prostate, testis, and skeletal muscle, highlighting the integrated prostate–testis–muscle axis underlying male reproductive and metabolic aging. Arrows indicate the direction of interactions among the different components of the integrated network. Created in BioRender. Bossio, S. (2026) https://BioRender.com/br9lpq3 (accessed on 14 February 2026).

Overall, these interventions target key pathways involved in inflammaging, including redox imbalance, mitochondrial dysfunction, impaired autophagy, and chronic inflammation. By modulating these mechanisms, they may influence both male reproductive aging and systemic phenotypes such as sarcopenia (Figure 6).

## 6. Discussion

### 6.1. Systemic Inflammaging of the Male Reproductive System

Male reproductive senescence, rather than being an isolated organ-specific phenomenon, may be conceptualized as a systemic manifestation emerging from a complex biological network. This process is thought to be driven by the interplay of chronic low-grade inflammation, immune remodeling, metabolic dysfunction, and endocrine imbalance. Within this framework, prostate and testicular aging emerge as interconnected manifestations of inflammaging rather than isolated organ-specific processes [1,4]. In this context, oxidative stress is considered an integral component of inflammaging, reinforcing immune–metabolic dysregulation and contributing to systemic aging phenotypes such as sarcopenia and metabolic decline. Together, these processes provide a coherent framework linking local tissue vulnerability to systemic aging phenotypes [8,84].

Importantly, the key conceptual advance emerging from this review is the recognition of male reproductive aging as a system-level phenomenon, in which the prostate, testis, and skeletal muscle are interconnected through a shared immune–metabolic and epigenetic network driven by inflammaging.

Persistent inflammatory signaling contributes to age-related prostate tissue remodeling and increased disease susceptibility [2,28]. The identification of proliferative inflammatory atrophy as a pre-neoplastic lesion further reinforces the concept that long-standing inflammation is not merely a bystander but is thought to contribute actively of prostate disease progression [40,41]. From a translational perspective, these observations suggest that interventions aimed at dampening inflammatory tone and restoring immune–metabolic balance may represent effective strategies to reduce prostate vulnerability and cancer risk before overt pathological transformation occurs [27].

Similar mechanisms characterize testicular aging, where inflammaging progressively compromises both spermatogenic and endocrine functions. In the testis, inflammaging has been associated with both spermatogenic impairment and endocrine dysfunction, partly through inflammatory remodeling of the testicular microenvironment and activation of pathways such as the NLRP3 inflammasome [57,124,125]. Importantly, these processes appear to be at least partially reversible, highlighting the potential of early systemic interventions to preserve testicular homeostasis during aging.

Outside the reproductive organs, inflammaging also affects skeletal muscle, positioning sarcopenia as a key systemic manifestation of aging [8,126]. In men, the bidirectional relationship between androgen deficiency, muscle loss, and inflammatory signaling establishes a self-reinforcing cycle that accelerates both reproductive and systemic aging [80,81]. This interdependence highlights skeletal muscle as both a target and a modulator of systemic inflammaging, with important implications for integrated therapeutic strategies.

### 6.2. Epigenetic and Senescence-Associated Mechanisms

An additional level of complexity in male reproductive aging is provided by epigenetic remodeling, which acts as a molecular interface between chronic inflammation, metabolic stress, and long-term alterations in gene regulation [65,66]. Emerging evidence indicates that epigenetic remodeling may act as a molecular memory of inflammaging, stabilizing stress-responsive transcriptional programs even after resolution of inflammatory stimuli. This mechanism provides a biological substrate through which chronic inflammation exerts sustained effects on tissue homeostasis and disease susceptibility [16]. In this context, epigenetic drift and inflammation-driven chromatin remodeling may contribute to sustained tissue vulnerability, increased disease susceptibility, and impaired regenerative capacity across the male reproductive system [69,72]. Importantly, epigenetic remodeling also provides a mechanistic framework through which nutritional and metabolic cues may influence inflammaging. Nutrient availability and cellular metabolic state directly regulate chromatin-modifying enzymes, linking dietary patterns, energy sensing, and inflammatory signaling to long-lasting changes in gene expression. Thus, epigenetic mechanisms act not only as passive records of inflammatory stress but also as dynamic and potentially reversible regulators of tissue vulnerability.

In addition to epigenetic remodeling, chronic oxidative stress may induce SIPS, thereby converting transient inflammatory and redox insults into stable alterations of cellular behavior. The acquisition of a SASP sustains a pro-inflammatory and tissue-remodeling milieu, reinforcing immune activation and stromal dysfunction over time. Emerging evidence further suggests that senescence-associated transcriptional instability, including alternative splicing events affecting stress-response and survival-related genes, may represent an additional layer of molecular memory that parallels epigenetic reprogramming. Collectively, these mechanisms provide a biological basis for the persistence of inflammaging-related tissue vulnerability beyond the initial stressor [127].

### 6.3. Lifestyle and Metabolic Interventions Targeting Inflammaging

This epigenetic-metabolic interface further supports the concept that inflammaging-related alterations in the male reproductive system are, at least in part, biologically modifiable through targeted lifestyle and metabolic interventions [74,79].

Non-conventional therapies and lifestyle interventions emerge as particularly attractive approaches due to their ability to modulate multiple aging-related pathways simultaneously. Nutraceuticals with pleiotropic metabolic and mitochondrial effects, caloric restriction and its mimetics, and lifestyle-based interventions converge on shared molecular mechanisms regulating inflammation, energy sensing, autophagy, and endocrine signaling [99,112,114,115]. Rather than acting as isolated treatments, these strategies may exert synergistic effects on the inflammaging network, offering a systems-level opportunity to attenuate age-related decline across tissues.

Lifestyle interventions targeting sarcopenia, including resistance exercise, adequate protein intake, and optimization of vitamin D status, hold particular translational relevance. In addition to their established effects on muscle mass and function, these interventions reduce systemic inflammatory burden, improve metabolic flexibility, and promote endocrine balance, thereby indirectly supporting prostate and testicular homeostasis [118,119,120]. In this context, the concept of an integrated prostate–testis–muscle axis provides a translational framework through which lifestyle-based strategies can be viewed as biologically grounded interventions influencing male reproductive aging trajectories. From a translational perspective, this integrated model suggests that interventions targeting systemic inflammation and metabolic dysfunction may influence multiple components of male reproductive aging. This supports the rationale for multimodal strategies combining lifestyle, metabolic, and nutraceutical approaches.

From this perspective, inflammaging should not be interpreted solely as an unavoidable consequence of chronological aging but rather as a biologically modifiable process. However, this concept requires cautious interpretation, as current evidence indicates that lifestyle and metabolic interventions primarily attenuate inflammatory signaling rather than fully reversing age-related physiological alterations. Accumulating evidence indicates that interventions such as caloric restriction and regular physical activity can modulate key inflammaging-related pathways, improving metabolic and redox homeostasis during aging [12,128]. For instance, long-term exercise has been shown to reduce systemic inflammatory markers in older adults, although profiles often remain distinct from those observed in younger individuals [128]. Accordingly, inflammaging appears to be modifiable, with its progression potentially slowed, rather than fully reversible.

## 7. Conclusions

Taken together, translational data argue that male reproductive aging should be understood as a systemic, interconnected process driven by “inflammaging” and metabolic musculoskeletal dysfunction, rather than just an isolated organ decline. While current research is limited by observational and preclinical data, it strongly supports an integrated model where lifestyle and metabolic interventions, such as diet and exercise, could be used to target and delay these age-related changes. These interactions may be monitored through representative biomarkers reflecting systemic crosstalk, including inflammatory cytokines (e.g., IL-6, TNF-α), metabolic indicators, and endocrine parameters such as circulating testosterone levels.

Core Takeaways

- A new System-Level Framework: Aging in the prostate, testes, and skeletal muscles is linked by systemic inflammation and immune–metabolic crosstalk.
- Promising Interventions: Modifying lifestyle through physical activity, caloric restriction, and nutraceuticals represents a viable strategy to preserve reproductive and metabolic health.
- Need for Further Study: Well-designed longitudinal studies and clinical trials are required to overcome current literature limitations and prove efficacy for these precision medicine interventions.

## Figures and Tables

**Table 1:** Lifestyle, metabolic, and nutraceutical interventions targeting pathways involved in inflammaging and the male reproductive system.

Intervention	Molecular Targets	Biological Effects	Potential Impact on Male Reproductive Aging
Caloric restriction	AMPK, SIRT1, mTOR	Improved mitochondrial function, enhanced autophagy, reduced systemic inflammation	Preservation of Leydig cell function, improved spermatogenesis
Resistance exercise	Myokine signaling, inflammatory cytokines	Reduced systemic inflammation, improved metabolic flexibility	Support of hormonal balance and muscle–reproductive axis
Adequate protein intake	mTOR signaling, muscle protein synthesis	Prevention of anabolic resistance and sarcopenia	Indirect support of reproductive and endocrine health
Vitamin D optimization	Vitamin D receptor signaling	Modulation of immune responses and hormonal pathways	Improved muscle function and potential androgen regulation
Nutraceutical supplementation (ALA, polyphenols, CoQ10)	Redox balance, mitochondrial pathways, NF-κB signaling	Reduction in oxidative stress and chronic inflammation	Improved sperm parameters and reproductive function

## Data Availability

No new data were created or analyzed in this study. Data sharing is not applicable to this article.
